# Acute-on-chronic liver failure triggered by postpartum variceal bleeding in a patient with alcohol-associated liver disease

**DOI:** 10.1007/s12328-026-02307-2

**Published:** 2026-03-18

**Authors:** Yoshiaki Kobayashi, Takefumi Kimura, Tomoo Yamazaki, Takanobu Iwadare, Kosuke Sonoda, Yasuhiro Tanaka, Tadanobu Nagaya

**Affiliations:** 1 Division of Gastroenterology, Department of Medicine, Shinshu University School of Medicine, 3-1-1 Asahi, Matsumoto, Nagano 390-8621 Japan; 2 Consultation Center for Liver Diseases, Shinshu University Hospital, Matsumoto, Japan; 3 Institute for Biomedical Sciences, Research Cluster for Social Implementation, Matsumoto, Japan; 4 Division of Nephrology, Department of Medicine, Shinshu University School of Medicine, Matsumoto, Japan; 5 Division of Obstetrics and Gynecology, Department of Medicine, Shinshu University School of Medicine, Matsumoto, Japan

**Keywords:** Acute-on-chronic liver failure, Alcohol-associated liver disease, Alcoholic hepatitis, Metabolic dysfunction-associated steatotic liver disease, Cesarean section, Postpartum

## Abstract

Pregnancy in women with cirrhosis carries substantial maternal risk, with variceal bleeding being one of the leading causes of mortality. Acute-on-chronic liver failure (ACLF) is a distinct clinical entity, yet cases triggered by postpartum variceal bleeding are uncommon. We describe a 41-year-old woman with alcohol-associated cirrhosis who conceived through in vitro fertilization and had no varices on endoscopy performed one year before delivery. On postpartum day 4 after cesarean section, she developed massive hematemesis from newly developed esophageal varices, culminating in ACLF with grade 4 hepatic encephalopathy and coagulopathy. Endoscopic variceal ligation successfully controlled the hemorrhage, while therapeutic plasma exchange and hemodiafiltration were required to manage hepatic encephalopathy. She ultimately made a complete recovery without liver transplantation. This case underscores the immediate postpartum period as a uniquely vulnerable window in women with cirrhosis, during which rapid hemodynamic shifts may precipitate variceal rupture and ACLF. It also suggests that repeat endoscopic screening during pregnancy may be warranted for selected high-risk patients.

## Introduction

Pregnancy with chronic liver disease carries substantial maternal and fetal risks. In cirrhotic patients, the likelihood of hepatic decompensation during pregnancy is markedly elevated, manifesting as ascites, variceal bleeding, or encephalopathy, and is often accompanied by cholestasis and the need for intensive care unit admission [1]. Among these complications, variceal bleeding is a leading cause of maternal mortality and necessitates vigilant risk assessment and management during pregnancy [2].

Acute-on-chronic liver failure (ACLF) is a syndrome defined by acute decompensation in patients with underlying chronic liver disease or cirrhosis, resulting in hepatic and extrahepatic organ failure and high short-term mortality [3, 4]. The acute insults commonly include alcohol abuse, bacterial infection, disease exacerbation, and gastrointestinal hemorrhage [5, 6].

Here, we report a rare case of ACLF precipitated by postpartum esophageal variceal bleeding after cesarean section in a patient with alcohol-associated liver disease (ALD). This case highlights how pregnancy-related hemodynamic changes may worsen portal hypertension and trigger acute decompensation in cirrhosis.

### Case report

A 41-year-old woman with long-standing heavy alcohol consumption (approximately 500 g/week since her early twenties) had first been noted to have liver dysfunction at age 25, diagnosed as hepatic steatosis. She developed hypertension at age 35, but despite persistently abnormal liver tests, she did not seek further evaluation.

**Fig. 1:**
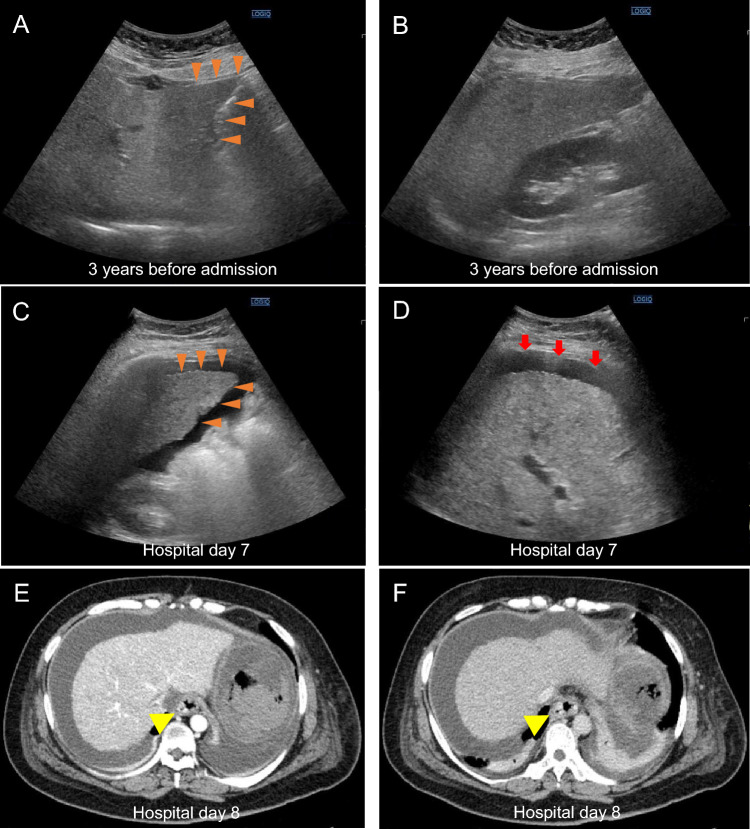
Abdominal ultrasonography and contrast-enhanced computed tomography findings. **A**, **B** Ultrasonography three years before admission: **A** Blunted liver edge with an irregular surface (arrowheads). **B** Hepatorenal contrast showing increased hepatic echogenicity, consistent with hepatic steatosis. **C**, **D** Ultrasonography at referral (hospital day 7): **C** Irregular liver surface (arrowheads) with coarse parenchymal echotexture. **D** Moderate ascites (arrows) without evidence of hepatic malignancy. **E**, **F** Contrast-enhanced CT on hospital day 8: Dilated vessels in the lower esophagus (arrowheads) without active bleeding

At age 38, during preconception counseling, laboratory tests again showed elevated aminotransferases (aspartate aminotransferase [AST], 120 U/L, and alanine aminotransferase [ALT], 60 U/L). Ultrasonography demonstrated hepatic steatosis with an irregular liver surface (Fig. 1A, B), and viral and autoimmune liver diseases were excluded. Based on her long-standing alcohol use and these findings, alcohol-associated liver disease (ALD) was diagnosed. Non-invasive fibrosis assessment indicated moderate fibrosis, and she was advised to abstain from alcohol. At age 39, after failing to reduce alcohol intake, she was referred to our department for worsening liver dysfunction. At age 40, esophagogastroduodenoscopy (EGD) showed no esophageal varices, and she subsequently conceived through in vitro fertilization. Her pre-pregnancy body mass index was 24.8 kg/m^2^, and she successfully abstained from alcohol during pregnancy, which was complicated by hypertension. During pregnancy, the patient was followed by the obstetrics team with biweekly visits. After alcohol abstinence, aminotransferase levels (AST ~ 40 U/L, ALT ~ 20 U/L), total bilirubin (~ 1.0 mg/dL), and platelet counts (~ 14.0 × 10^4^/μL) remained stable. However, hepatic imaging evaluations were not performed during pregnancy.

She was admitted for threatened preterm labor and underwent emergency cesarean section on hospital day 4 at 31 weeks and 3 days for superimposed preeclampsia with thrombocytopenia (platelet count approximately 8.0 × 10^4^/μL), without evidence of hemolysis (including normal lactate dehydrogenase levels) or marked elevation of aminotransferases, with an estimated intraoperative blood loss of approximately 1000 mL. The neonate had transient tachypnea. In the early postoperative period, ascites worsened and liver dysfunction persisted (total bilirubin 3.08 mg/dL, and prothrombin time [PT] 53%). She was referred to our department on hospital day 7. At presentation, she was alert but required high-flow nasal oxygen therapy (60 L/min) to maintain an SpO₂ of 93%. Physical examination revealed mild jaundice, abdominal distension, and bilateral leg edema. Laboratory results showed elevated liver enzymes, low albumin (2.4 g/dL), prolonged PT (64.6%), and markedly increased fibrosis markers (FIB-4 2.27, mac-2 binding protein glycosylation isomer 6.76 AU/mL, autotaxin 2.397 mg/L, type IV collagen 7S 39.7 ng/mL) (Table 1). Ultrasonography indicated cirrhosis with moderate ascites but no malignancy or portal vein thrombosis (Fig. 1C, D). Her Child–Pugh score was 11 (class C) and MELD score 12.

**Table 1:** Laboratory data at referral to our department (hospital day 7)

Hematology			Biochemistry			Autoimmune antibodies	
WBC	**27,810**	/μL	Alb	**2.4**	g/dL	ANA	(–)
Neu	**76.0**	%	AST	**44**	U/L	AMA-M2	(–)
Lym	16.0	%	ALT	26	U/L		
RBC	**345 × 10** ^**4**^	/μL	ALP*	**358**	U/L		
Hemoglobin	**11.1**	g/dL	γ-GTP	33	U/L		
Platelets	**15.6 × 10** ^**4**^	/μL	T-Bil	**2.08**	mg/dL		
Coagulation			LDH	**351**	U/L		
PT	**64.6**	%	BUN	**21.3**	mg/dL		
PT-INR	**1.28**		Cre	0.59	mg/dL		
APTT	31.6	sec	Na	**135**	mEq/L		
FIBG	**483**	mg/dL	K	4.0	mEq/L		
AT-III	**59.6**	%	CRP	**11.29**	mg/dL		
			M2BPGi	**6.76**	AU/mL		
			Autotaxin	**2.397**	mg/L		
			**COL4-7S**	**39.7**	ng/mL		

Bold values indicate abnormal results.

Alb, albumin; ALP, alkaline phosphatase; ALT, alanine aminotransferase; AMA-M2, anti-mitochondrial antibody type M2; ANA, anti-nuclear antibody; APTT, activated partial thromboplastin time; AST, aspartate aminotransferase; AT-III, antithrombin III; BUN, blood urea nitrogen; COL4-7S, type IV collagen 7S; Cre, creatinine; CRP, c-reactive protein; FIBG, fibrinogen; γ-GTP, gamma-glutamyl transpeptidase; K, potassium; LDH, lactate dehydrogenase; Lym, lymphocytes; M2BPGi, mac-2 binding protein glycosylation isomer; Na, sodium; Neu, neutrophils; PT, prothrombin time; PT-INR, prothrombin time-international normalized ratio; RBC, red blood cells; T-Bil, total bilirubin; WBC, white blood cells.

*ALP was measured using the International Federation of Clinical Chemistry (IFCC) method.

**Fig. 2:**
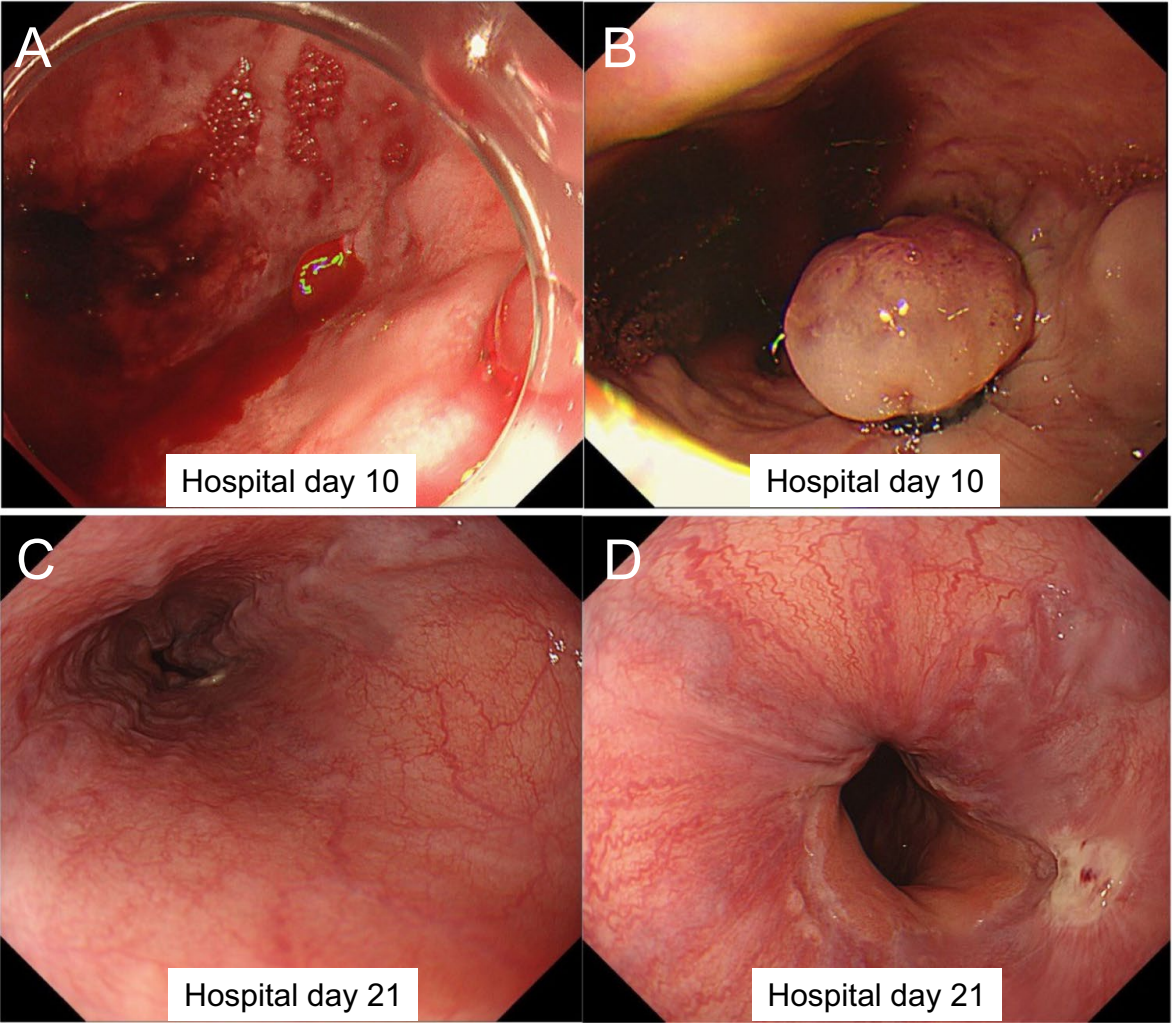
Esophagogastroduodenoscopy. **A**, **B** Endoscopy on hospital day 10 showing active bleeding from lower esophageal varices, with hemostasis achieved by endoscopic variceal ligation. **C**, **D** Follow-up endoscopy on hospital day 21 showing no lesions requiring treatment (Li, F1, Cb, RC0)

On hospital day 8, she developed hematemesis. Computed tomography (CT) revealed dilated esophageal vessels (Fig. 1E, F), and emergent EGD showed actively bleeding newly developed esophageal varices, which were treated with endoscopic variceal ligation (Fig. 2A, B). On hospital day 10, she developed agitation followed by grade 4 hepatic encephalopathy, with ammonia rising to 138 μg/dL (Table 2). She fulfilled the EASL–Chronic Liver Failure Consortium criteria for ACLF with brain failure and respiratory failure, consistent with the severity of ACLF grade 2 [7, 8]. Comprehensive infection screening to identify potential infectious triggers of ACLF was negative.

**Table 2:** Laboratory data at the time of ACLF diagnosis (hospital Day 10)

Hematology			Biochemistry		
WBC	**45,180**	/μL	Alb	**2.6**	g/dL
Neu	68.0	%	AST	**69**	U/L
Lym	**12.0**	%	ALT	36	U/L
RBC	**258 × 10** ^**4**^	/μL	ALP*	**211**	U/L
Hemoglobin	**8.2**	g/dL	γ-GTP	36	U/L
Platelets	19.9 × 10^4^	/μL	T-Bil	**3.49**	mg/dL
Coagulation			LDH	**492**	U/L
PT	**43.6**	%	BUN	**50.0**	mg/dL
PT-INR	**1.73**		Cre	0.79	mg/dL
APTT	30.6	sec	Na	144	mEq/L
FIBG	268	mg/dL	K	4.4	mEq/L
AT-III	**54.2**	%	CRP	**4.02**	mg/dL
			NH3	**138**	μg/dL

Bold values indicate abnormal results

Alb, albumin; ALP, alkaline phosphatase; ALT, alanine aminotransferase; APTT, activated partial thromboplastin time; AST, aspartate aminotransferase; AT-III, antithrombin III; BUN, blood urea nitrogen; Cre, creatinine; CRP, c-reactive protein; FIBG, fibrinogen; γ-GTP, gamma-glutamyl transpeptidase; K, potassium; LDH, lactate dehydrogenase; Lym, lymphocytes; Na, sodium; Neu, neutrophils; NH3, ammonia; PT, prothrombin time; PT-INR, prothrombin time-international normalized ratio; RBC, red blood cells; T-Bil, total bilirubin; WBC, white blood cells.

*ALP was measured using the International Federation of Clinical Chemistry (IFCC) method

**Fig. 3:**
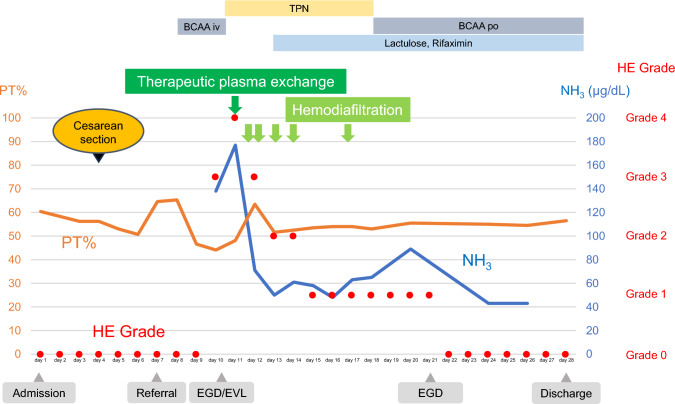
Clinical course of the patient. BCAA, branched chain amino acids; EGD, esophagogastroduodenoscopy; EVL, endoscopic variceal ligation; HE grade, hepatic encephalopathy grade; iv, intravenous; NH_3_, ammonia; po, per oral; PT, prothrombin time; TPN, total parenteral nutrition

On hospital day 11, she remained comatose with ammonia 177 μg/dL. Brain CT was unremarkable. Therapeutic plasma exchange and hemodiafiltration were initiated to manage encephalopathy and prevent multi-organ failure, while liver transplantation remained a contingency. Her neurological status improved progressively—grade 3 on day 12, grade 1 on day 15, and full recovery by day 22. Ammonia levels and ascites decreased, and hemodiafiltration was discontinued on hospital day 17. Her respiratory status gradually improved, and supplemental oxygen was discontinued. Follow-up EGD on hospital day 21 showed small residual varices without high-risk stigmata (Li, F1, Cb, RC0) (Fig. 2C, D). She was discharged home on hospital day 28 with reinforcement of strict alcohol abstinence and psychiatric support (Fig. 3). The neonate recovered well and was discharged on day 60.

## Discussion

This case illustrates ACLF precipitated by variceal bleeding in the immediate postpartum period after cesarean section in a patient with alcohol-associated cirrhosis. To our knowledge, this is the first reported case of postpartum ACLF following cesarean delivery. HELLP syndrome (hemolysis, elevated liver enzymes, and low platelet count) and acute fatty liver of pregnancy were considered but excluded on the basis of the absence of hemolysis (including normal lactate dehydrogenase levels) and the absence of any elevation of aminotransferases. These findings supported a diagnosis of ACLF in the setting of underlying cirrhosis.

Pregnancy-related hemodynamic and physiological changes can precipitate acute decompensation in cirrhotic patients. ACLF is characterized by acute deterioration of chronic liver disease with hepatic and extrahepatic organ failure and high short-term mortality [9]. Common precipitants include bacterial infection, alcohol-related hepatitis, drug-induced liver injury, viral reactivation, and gastrointestinal hemorrhage [10]. In this case, the direct trigger was variceal bleeding, but an important question is why rupture occurred specifically in the immediate postpartum period.

Although this patient had ALD, she had no prior history of varices, and EGD performed one year earlier was negative. During pregnancy, marked hemodynamic changes—including increased plasma volume and cardiac output with reduced systemic vascular resistance—worsen portal hypertension and facilitate the development of varices [11]. After delivery, withdrawal of placental vasodilators increases systemic vascular resistance [12], and cardiac output further rises during labor and the early postpartum period [13]. Elevated portal venous flow persists for several weeks [14], with hemodynamics returning to baseline only after approximately two weeks [15]. These dynamic changes likely created a vulnerable window in which newly developed varices became prone to rupture, thereby serving as an important contributing factor to the subsequent development of ACLF.

In addition to these hemodynamic shifts, perioperative stress may have further reduced hepatic reserve. The emergency cesarean section was associated with an estimated intraoperative blood loss of approximately 1000 mL. Although this volume is not uncommon in obstetric practice, it represents a significant physiological insult in patients with cirrhosis, particularly in the presence of thrombocytopenia and impaired coagulation, and may have contributed to hepatic decompensation.

Esophageal variceal bleeding is a well-recognized cause of maternal morbidity in women with cirrhosis. Endoscopic assessment should ideally be performed before conception to guide prophylactic management of varices; however, EGD can also be safely performed during pregnancy when clinically indicated [16, 17]. Preventive therapy with non-selective β-blockers or endoscopic ligation should be considered for medium-to-large varices. In the present case, despite the absence of varices one year before pregnancy, bleeding varices developed rapidly during the peripartum period. The absence of varices before conception and stable liver biochemistry throughout pregnancy underscore the limitations of current risk stratification and the difficulty in predicting rapid progression of portal hypertension in cirrhotic pregnancy. Although robust evidence is lacking, this case suggests that careful clinical and imaging-based monitoring for worsening portal hypertension during pregnancy may be a reasonable approach.

This case also highlights the potential benefit of therapeutic plasma exchange and hemodiafiltration for ACLF. Therapeutic plasma exchange improves coagulopathy through replacement with fresh frozen plasma and may reduce bleeding risk [18]. This hemostatic support is particularly relevant for postpartum patients who are prone to bleeding complications. Hemodiafiltration allows removal of hepatotoxic substances while maintaining hemodynamic stability [19]. Combined therapy has been reported to improve survival in patients awaiting transplantation or ineligible for transplantation [20], consistent with the favorable response observed in our patient.

In summary, this patient with alcohol-associated cirrhosis developed ACLF due to variceal bleeding shortly after cesarean section, likely triggered by pregnancy-related hemodynamic shifts that exacerbated portal hypertension. These findings underscore the need for timely variceal screening and multidisciplinary management in pregnant women with chronic liver disease.
